# The Function of Posterior Middle Temporal Gyrus in Conceptual Expansion

**DOI:** 10.1002/pchj.70025

**Published:** 2025-06-29

**Authors:** Jingjing Yang, Ziyi Li, Ze Zhang, Jing Luo

**Affiliations:** ^1^ Beijing Key Laboratory of Learning and Cognition, School of Psychology Capital Normal University Beijing China; ^2^ State Key Laboratory of Cognitive Neuroscience and Learning & IDG/McGovern Institute for Brain Research Beijing Normal University Beijing China; ^3^ Department of Psychology Shaoxing University Shaoxing China

**Keywords:** alternate uses task, conceptual expansion, creativity, middle temporal gyrus

## Abstract

Conceptual expansion, referred to as the expansion of existing conceptual structures and the generation of new concepts, is a key cognitive component of creative ideation in human beings. However, the specific brain regions associated with the process of conceptual expansion remain unclear, particularly in the field of divergent thinking. In the present study, we examined neural correlates of conceptual expansion in the alternate uses task (AUT). Specifically, during functional magnetic resonance imaging (fMRI) scanning, participants were asked to process a set of creative AUT ideas, each consisting of a common object and a corresponding alternate use, and then in the post‐scan phase, they were required to evaluate the degree of conceptual expansion for each AUT idea (i.e., compared with the familiar concept, the extent to which the AUT idea could expand the conceptual boundaries of the object). By linking the behavioral assessments with brain activation, the results showed that greater engagement of the posterior middle temporal gyrus (pMTG) was involved in the processing of creative AUT ideas with higher conceptual expansion, which aligns with previous findings from other creative domains such as insight problem solving and creative product design. Given the recognized function of the pMTG in conceptual processing, our findings indicate that the pMTG may specifically support the forming of new conceptual categories in AUT.

## 1 Introduction

Creative ideation of human beings entails not only the formation of new associations, but also the expansion of old concepts and the generation of new categories, which differs from the animals' novel problem‐solving behaviors that have creativity‐like features (Shettleworth 2012; Kaufman and Kaufman 2004; Kaufman et al. 2011). Sociologist Max Weber once said “man is an animal suspended in webs of significance he himself has spun” (Weber 2001). In contrast to other animals, there is highly developed language system in human beings that is capable of understanding, creating, and spreading abstract and complex concepts. Conceptual expansion refers to the cognitive operation on existing concepts in creative thinking, that is, widening and augmenting the boundaries of known concepts to include new attributes or features thereby forming new concepts (Abraham et al. 2012, 2018; Abraham and Windmann 2007; Abraham 2014; Ward 2008, 1994; Ward et al. 1997). This process is unique to human creativity. That is, for humans, creativity means not only the accidental formation of certain new associations (Mednick 1962), but also the generation of “new nodes” or “concepts” in the “webs of significance”. For example, in the field of product and technology, the invention of electric vehicles updates the traditional concept that fuel power is the essential feature of cars, and the invention of digital cameras overturns the fundamental thought that the essential attribute of the camera is that it must capture images with film. These creations were accompanied by the emergence of a conceptual system that included additional properties and transcended the common knowledge of the time, significantly advancing human civilization. Moreover, various creative theories have explicitly or implicitly positioned operation on concepts as a critical component of creative thinking. For instance, an early creativity researcher, Rhodes, who is the proponent of Four P's theory, said “The word creativity is a noun naming the phenomenon in which a person communicates a new concept (which is the product)” (Rhodes 1961, p. 305). In addition, Glăveanu, who proposed the Five A's framework, recognized the function of societal scripts, wherein the processing of concepts occurs in weaving the five basic elements of creativity (actor, action, artifact, audience and affordances) together (Glaveanu 2013). In the propulsion model of creativity proposed by Sternberg and colleagues, besides replication and redefinition, which directly involve the operation on concepts in a specific domain, integration, redirection, reconstruction and reinitiation also imply the adjustment, recombination, and even complete replacement of existing concepts (Sternberg 1999; Sternberg et al. 2001). That is, the ability to understand, retain, and practice the new concepts is essential for human innovation and creation. Thus, in this study we sought to investigate the process of expanding existing concepts (i.e., generating new concepts) involved in the creative thinking.

Previous studies have adopted some approaches to assess conceptual expansion (Abraham et al. 2012, 2018). For example, in the original conceptual expansion task, participants were required to imagine and draw an animal that lives on another plane. The task assessed how far one person's depiction of an animal deviates from general schema of animals on Earth (Ward 1994; Ward et al. 1997; Birdsell 2019). That is, conceptual expansion engages the inhibition of stereotypical instances to elaborate and expand the structure and attributes of concepts. The more an individual can imagine an animal having unusual features, such as bilateral asymmetry and the absence of common appendages or sense organs (e.g., wheels in place of feet), the greater one's conceptual expansion (Abraham et al. 2005, 2006). Similarly, in the alternate uses task (AUT), generating new uses for a common object requires the expansion of the conceptual structure of the object to include previously unassociated features or exemplars (Abraham and Windmann 2007; Abraham 2014; Ward 2008). For example, the typical use of a shoe is to protect the foot, then an alternate use of the shoe could be as a plant pot, in which the shoe may not be fully considered as footwear, and it has been classified as a container for flowers. That is, the original definition of the shoe was broadened to include new attributes, generating an expanded new concept.

While several studies have examined the brain activation related to conceptual expansion, the neural correlates of conceptual expansion involved in typical creative thinking such as AUT are poorly understood. Specifically, previous studies have consistently posited that the entire processing of creative metaphor or AUT idea inherently involves the expansion of existing conceptual structures and the addition of new elements (Kröger et al. 2012, 2013; Rutter, Kröger, Stark, et al. 2012; Rutter, Kröger, Hill, et al. 2012; Abraham et al. 2021). For example, these studies asked participants to view some AUT ideas (e.g., shoes‐plant pot) or metaphors (e.g., the clouds have danced over the city), and contrasted the novel and useful (NU) ideas with the novel and useless (NUs) and familiar and useful (FU) ideas as control conditions to examine the neural signature of conceptual expansion. However, what these comparisons detect might be brain regions associated with the processing of novelty and usefulness (NU–FU = novelty and NU–NUs = usefulness) and less specific to conceptual expansion. In their studies, conceptual expansion generally is equivalent to all cognitive operations related to a certain group of creative items. However, conceptual expansion might only be a cognitive component in creative ideation, and a more precise experimental design is needed to obtain neural correlates of conceptual expansion.

Indeed, the performance in creative tasks is interrelated with the degree of conceptual expansion. Some studies have shown that in the actual generation of creative ideas, conceptual expansion was correlated with creative performance. That is, for one individual, more creative answers in the tasks might signify greater deviation from the original concepts (Ward 1994; Abraham and Windmann 2007; Benedek et al. 2020; Beaty et al. 2020). However, as a cognitive component or operation of creative thinking, some brain regions related to creative thinking may specifically mediate the process of conceptual expansion. In the passively processing of creative materials (Kröger et al. 2012, 2013; Abraham et al. 2012, 2018, 2021), there may be varying degrees of conceptual expansion. Therefore, the present study aimed to identify the brain regions specifically associated with conceptual expansion by utilizing a set of creative AUT ideas (i.e., a common object paired with an alternate use) and differentiating the relative degree of conceptual expansion for each participant. Specifically, we asked participants to process these AUT ideas during functional magnetic resonance imaging (fMRI) scanning, and then in the post‐scan phase, they were required to evaluate the degree of conceptual expansion for each AUT idea (i.e., compared with the familiar concept, the extent to which the AUT idea could expand the conceptual boundaries of the object) on a 6‐point scale. We performed the contrast between the processing of creative AUT ideas, which were subjectively judged as having a high and low degree of conceptual expansion. Moreover, we also took the creativeness scores of the AUT ideas as a covariate of no interest to control for the creativity of materials.

Previous neuroimaging studies have found the involvement of the posterior middle temporal gyrus (pMTG) in the generation of new concepts. For instance, using creative product designs (e.g., a lid with an L‐shaped handle that can stand stably on the table), Ren et al. (2020) found that neural activity patterns in the MTG were consistent with the behavioral assessments of new concept generation, suggesting that the MTG supports the formation of new concepts or categories in creative thinking (Ren et al. 2020, 2023; Zhang et al. 2020). Some studies proposed that patients with MTG damage, especially with posterior damage, have conceptual classification or categorical processing deficits (Hodges et al. 2000; Chao et al. 1999; Brambati et al. 2006). Besides, studies of insight problem solving also found that compared with the answers to conventional knowledge questions (CKQ) as a control condition, the presence of solutions for constraint relaxation riddles (CRR) that explicitly involved the expansion of conceptual boundaries engaged greater activation of the pMTG (Zhang et al. 2021). These studies suggest that the posterior part of the temporal lobe located in the ventral pathway might participate in basic conceptual categorical representation, mediating the expansion of conceptual properties in creative thinking. Therefore, in the current study, by relating behavioral assessments to brain activation, we hypothesized that the pMTG would be recruited in the high degree of conceptual expansion.

## 2 Methods

### 2.1 Participants

Thirty‐four right‐handed healthy university students (15 males, mean age ± SD, 23.06 ± 2.17) with normal or corrected‐to‐normal vision participated in the study. All participants reported no history of neurological disorders. Written informed consent was obtained from all participants before the fMRI scanning, and all the experimental protocols were approved by the ethics committee of the Center for Biomedical Imaging Research, Tsinghua University. Four participants' data were not included in the final formal analysis because of an insufficient number of trials (less than 15) in certain conditions.

### 2.2 Task and Stimuli

**FIGURE 1:**
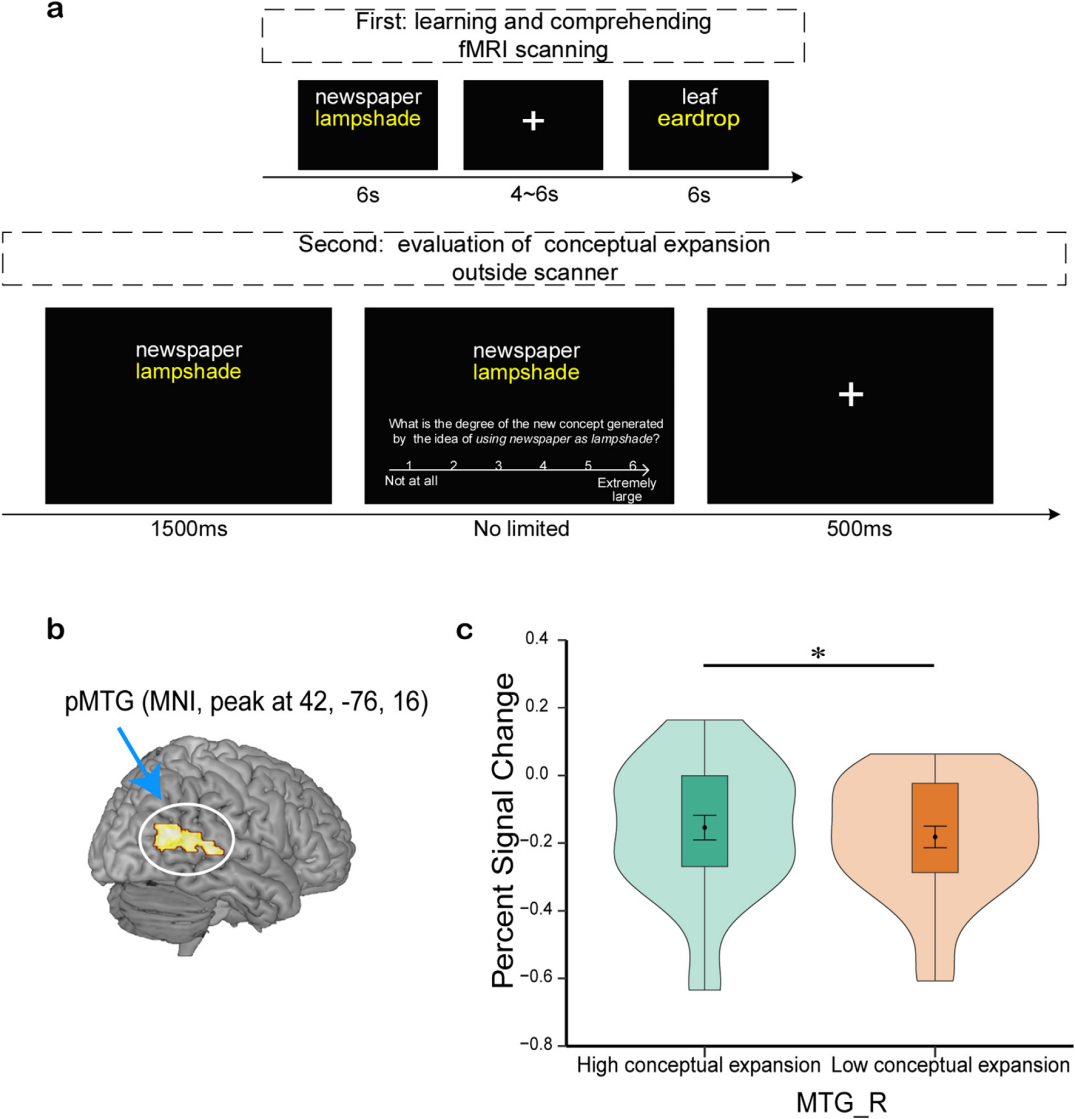
Experimental procedure and results. (a) The experiment consisted of two phases. (b) Right pMTG (MNI, peak at 42, −76, 16) is involved in high degree of conceptual expansion in contrast to low conceptual expansion condition. (c) Violin graphs represent functional activation in the defined pMTG ROI (10‐mm sphere centered at the peak of the pMTG cluster (*xyz* = [44, −72, 22]) reported in the Ren et al. (2020)‘s study), showing greater engagement during higher conceptual expansion. *p < 0.05; MNI, Montreal Neurological Institute; pMTG, posterior middle temporal gyrus; R, right.

These AUT ideas were selected from a database of our lab (Table S1). The formal experiment consisted of 100 readily understandable creative AUT ideas (each common object paired with an creative usage) and 20 filled stimuli (different objects paired with non‐creative usages, such as using the compass to draw a circle or using the Post‐it note to catch mice). During the fMRI scanning, a total of 120 trials were equally divided into four runs with 25 creative AUT ideas and five filled stimuli in each run. For each trial, the participants were shown an object and its usage for 6 s. Within this timeframe, they were instructed to focus on understanding the stimulus and evaluate whether the given usage of the object was creative or not. The filled stimuli were included only to control the participants' reaction tendency and ensure a “no” response, without contributing to the primary dataset. The presentation order of the trials was randomized in each run, and the sequence of the four runs was counter‐balanced across all participants. During scanning, to ensure signal stability and improve the signal‐to‐noise ratio, there was a 1 s fixation cross stage and a 4–6 s fixation cross stage before the presence of the first trial in each run, and there was a 15 s blank screen at the end of the run. Moreover, there was an inter‐trial interval with a fixation cross for 4–6 s between two trials, and a 1 min rest interval between the two functional runs (Figure 1a).

In the post‐scan phase, participants completed the conceptual expansion evaluation task in another quiet room. In this task, the 100 creative AUT ideas that were presented during the scanning were shown in a random order; participants were asked to subjectively evaluate the degree to which the AUT idea could expand the conceptual boundaries of the object (Ren et al. 2020). Specifically, in each trial, participants first viewed the AUT idea for 1500 ms and were required to evaluate the degree of conceptual expansion (e.g., compared with the familiar concept of *shoe*, what is the degree of the new conceptual category generated by the idea of using *shoe* as *plant pot*) on a 6‐point scale (1 = *not at all*, 6 = *extremely large*) (Figure 1a). The degree of generating a new conceptual category refers to compared with its familiar concept, the extent to which the alternate use expands existing object concepts to include novel features, and therefore widening its original structure and generating an expanded new concept. The participants were required to initially think of the existing conceptual information of the object, such as its prototypical color, size, shape, and function. Then they were instructed to consider that when the object was used for this new use, the degree to which it introduces certain new features for the object, redefining the object beyond the acquired conceptual category. The Cronbach's alpha of the measurement is 0.98. Before the beginning of each task, task‐related instructions were explained, and participants were familiarized with the experimental procedure and performed practice trials.

Based on each participant's evaluation of conceptual expansion for each creative AUT idea in the conceptual expansion evaluation task, the creative AUT ideas are sorted as low conceptual expansion trials (LCE, the rating score is equal and less than 3, that is the familiar conceptual category of the object is not redefined by the alternate use), or high conceptual expansion trials (HCE, the rating score is equal and larger than 4, that is the conceptual category of the object is restructured by the alternate use). The formal statistical analysis included the data of 30 participants. Specifically, four participants reported that the majority of AUT ideas greatly expanded the existing conceptual structure of the object, resulting in an insufficient number of trials in the LCE condition (*N* < 15), which may be due to all the AUT ideas presented in the experiment being creative and expanding the original conceptual definition of the object to a certain extent. The details of MRI data acquisition and analysis are provided in Table S2.

## 3 Result

In the within‐subject contrast, for the 30 participants, the number of trials in the HCE condition ranges from 15 to 85, with an average of 49.3 (SD = 18.71, SE = 3.42), and the number of trials in the LCE condition ranges from 15 to 85, with an average of 50.7 (SD = 18.71, SE = 3.42). There is no significant difference between the number of trials in the two conditions (*t*_(29)_ = 0.195, *p* = 0.847), which may suggest that the contrast between these two conditions should be unbiased. The neural basis of conceptual expansion was examined by comparing the HCE against the LCE condition (HCE minus LCE), and increased activity was found in the right hippocampus, right pMTG, and left parahippocampus gyrus using a voxelwise threshold of uncorrected *p* < 0.005, cluster size ≥ 30 (Figure 1b; Table S3). Moreover, the pMTG peak remained significant after controlling for the creativity scores of the AUT ideas obtained in the pilot study, suggesting that the pMTG activation can be conceptual expansion‐specific and at least partially independent of the creativeness score. In addition, we also chose the peak of the pMTG region (*x*, *y*, *z* = 44, −72, 22), whose neural activity pattern was found to selectively represent conceptual expansion in a previous study using creative design (Ren et al. 2020), as the center of the region of interest (ROI) of a 10‐mm sphere, and utilizing paired *t* test, we again found significantly higher activation in the HCE relative to the LCE condition (*t*_(29)_ = 2.229, *p* = 0.034) (Figure 1c).

In addition to conducting a within‐subjects contrast of high versus low conceptual expansion, linear effects of conceptual expansion were further analyzed with a parametric analysis considering the scores of conceptual expansion for these AUT ideas. Specifically, we entered, trial by trial, a conceptual expansion score for each AUT idea obtained in the post‐scan task as a covariate of interest for each trial (i.e., regressors for conceptual expansion). We contrasted the modulatory effects of the conceptual expansion covariate against baseline at the first level. At the second level, we entered these first‐level contrast images into a random‐effect one‐sample t test. This analysis revealed that higher conceptual expansion was related to greater brain activation in the left (peak at −42, −64, and 12) and right pMTG (peak at 44, −68 and 16) (*p* < 0.005, uncorrected).

## 4 Discussion

Conceptual expansion is a cognitive component of creative ideation, which means widening the existing structure of the concept to include new attributes and therefore generating an expanded new concept. The present study investigated the neural basis of conceptual expansion in divergent thinking. By classifying the creative AUT ideas into high and low degrees of conceptual expansion, we found that compared with the AUT ideas with low conceptual expansion, the processing of the AUT ideas with high conceptual expansion involved greater pMTG activation. Given that the recognized function of pMTG in conceptual processing, these findings suggest that the pMTG region might play a key role in conceptual expansion in the field of divergent thinking.

Beyond previous studies, our study utilized creative AUT ideas and classified these AUT ideas into two different conceptual expansion conditions individually according to individual responses of each participant, which enables a more direct and strict estimation of neural responses associated with conceptual expansion. We observed enhanced involvement of pMTG in the processing of AUT ideas with a higher degree of conceptual expansion, providing evidence that the pMTG may specifically support the expansion of object concepts in an alternate uses task. That is, during the forging of new links between ordinary objects and their alternate uses, pMTG could contribute to the broadening of existing conceptual boundaries of the objects and the generating of new concepts (Ren et al. 2020, 2023; Zhang et al. 2020, 2021). The pMTG is known to be associated with the processing of conceptual categories in semantic tasks and responsible for coding semantic information of concepts, playing a vital role in the representation of conceptual content (Liuzzi et al. 2021; Fairhall and Caramazza 2013; Wei et al. 2012). Some studies further proposed that patients with posterior MTG damage have impaired cognitive function related to object classification or category processing (Hodges et al. 2000; Chao et al. 1999; Brambati et al. 2006). Thus, by linking behavioral assessments of conceptual expansion with brain activation, our findings demonstrate that the pMTG might specifically facilitate the formation of new conceptual categories in divergent thinking. Besides, considering the interrelation between creativity and conceptual expansion, to control the potential effect of creativity, we reran the contrast (HCE minus LCE) with the creativeness scores of the AUT ideas modeled as a covariate of no interest and found that the peak of pMTG remained significant. Thus, our results expand the role of pMTG in supporting conceptual expansion in creative design to divergent thinking and further suggest such engagement could be independent of creativity.

In addition, we also found the activation of the medial temporal lobe (i.e., left parahippocampal gyrus and right hippocampus) in the comparison of HCE and LCE conditions. Compared with the LCE condition, there was greater involvement of the hippocampus and parahippocampal gyrus in the processing of AUT ideas in the HCE condition. We suggest that the MTL plays a role in semantic integration and the construction of novel representations in the processing of AUT ideas. The MTL, especially the hippocampus, contributes to the construction of configural representations and supports the binding of stimulus features together to form a unified representation (Eichenbaum et al. 2007; O'Reilly and Rudy 2001; Eichenbaum 2004; Staresina and Davachi 2008, 2009). Previous studies have also found that the hippocampus is engaged in creative tasks, playing a crucial role in the formation of new links between individual elements (Luo and Niki 2003; Huang et al. 2015; Wu et al. 2019, 2021; Ren et al. 2020; Shen et al. 2017; Zhang et al. 2020). Therefore, in the present study, the extensive participation of the MTL demonstrates that when the objects were presented with alternate uses, participants bound them together to form an integral representation, while the greater activation of the MTL in the HCE condition may indicate an increased need for unitary representation construction in the processing of AUT ideas with a higher degree of conceptual expansion. That is, compared with the LCE condition, there may be greater difficulty in forming novel unified representations between separated elements in the HCE condition.

Moreover, in our study, we utilized a conceptual expansion evaluation task to measure the degree of conceptual expansion for AUT ideas in the post‐scan phase. The task is adapted from previous studies such as Ren et al. (2020), in which they used creative product design and asked participants to assess the extent to which each novel and appropriate design represents a new concept compared with the conventional object concept. In the present study, we asked each participant to subjectively assess the degree to which the alternate uses expand the concepts of objects for creative AUT ideas. For example, among these creative AUT ideas, the participants rated that the AUT idea of using *the shoelace* as *a tying cord for notepaper* could not or only minimally expand the conceptual structure of shoelace, and the AUT idea of using *the ping‐pong ball* as *a miniature potted plant* could largely restructure the familiar conceptual category of ping‐pong ball. We believed that in this task, the participants were aware of what was being evaluated and capable of evaluating effectively, and these ratings allowed us to distinguish the relatively different degrees of conceptual expansion for each participant. Then in the within‐subject contrast, we classified the AUT ideas presented during fMRI scanning into HCE and LCE conditions according to each participant's evaluation. The distribution of trial numbers in the two conditions was not very concentrated, which resembles the subsequent memory effect paradigm in the field of memory, where trial distribution between the two experimental conditions is typically asymmetrical, while maintaining a fixed total number of trials across the conditions. Thus, we believed that by comparing the HCE with LCE conditions, we could identify the brain regions associated with the differences in the degree of conceptual expansion. In this study, the results revealed in the whole‐brain analyses incline to be taken as exploratory due to the relatively lenient statistical thresholds, and future work could focus on the function of pMTG in the process of conceptual expansion in divergent thinking, employing different analysis methods and more conservative thresholds.

In sum, the present study utilized the subject‐determined trial classification to separate creative AUT ideas into two levels of conceptual expansion. The findings suggest that the processing of creative AUT ideas with higher conceptual expansion is associated with greater engagement of pMTG, a region that is sensitive to semantic categories and plays a critical role in the representation of conceptual content, indicating that pMTG supports the expansion and updating of conceptual categories in creative ideation to accomplish the creative progress of knowledge.

## Ethics Statement

All the experimental protocols were approved by the ethics committee of the Center for Biomedical Imaging Research, Tsinghua University, and informed written consent was obtained from all participants before the experiment.

## Conflicts of Interest

The authors declare no conflicts of interest.

## Supporting information


**Table S1.** Experimental stimuli in Detail.
**Table S2.** fMRI data acquisition and analyses in Detail.
**Table S3.** Brain regions associated with high conceptual expansion and low conceptual expansion.

## Data Availability

The data that support the findings of this study are available from the corresponding author upon reasonable request.
