# Supplementary material for: The Function of Posterior Middle Temporal Gyrus in Conceptual Expansion
Source: Psych J. 2025 Jun 29;14(5):758–64. doi: 10.1002/pchj.70025 (PMC12520840; doi:10.1002/pchj.70025)
Supplement: Supplementary file 1 — Table S1. Experimental stimuli in Detail. Table S2. fMRI data acquisition and analyses in Detail. Table S3. Brain regions associated with high conceptual expansion and low conceptual expansion. [file PCHJ-14-758-s001.doc]

**Table S1 Experimental stimuli in Detail**

| Section | Descriptions |
| --- | --- |
| Alternate use task materials | In the AUT database of our lab, there have been 339 common objects and each object corresponds to several uses (conventional or unconventional) collected from different cohorts of participants. Then, 10 raters in the pilot study who were graduate students of Psychology and familiar with the alternate use task were recruited to rate each usage in terms of novelty, usefulness, creativity, and understandability on a 5-point Likert scale, and the inter-rater reliability was 0.43 (ICC=0.43). Specifically, for the novelty dimension, the raters should consider the extent to which the use of the object is novel, original, unusual or uncommon; for the usefulness, they should focus on the extent to which the use of the object could be realizable and appropriate; for the creativity, it is necessary to consider whether it is a creative idea and the level of creativity; for the understandability, it refers to whether the description of the use of the object is clarity and comprehensibility. Moreover, for the formal experiment, 100 common objects were selected from 339 objects based on the pretest rating, and each object corresponds to a creative alternate use. The creativity scores of the 100 AUT ideas range from 3.0 to 4.0, yielding an average creativity score of 3.43 (SD = 0.27), with an average novelty score of 3.85 (min: 3.18, max: 4.65, SD=0.33), usefulness score of 3.51 (min: 3.06, max: 4.24, SD=0.31) and understandability score of 4.53 (min: 3.59, max: 4.82, SD=0.28). Furthermore, we have also selected 20 different common objects paired with a conventional use or novel but inappropriate use from the database as filled stimuli (including 10 familiar and useful AUT ideas and 10 novel but useless AUT ideas). Out of these filled stimuli, for the 10 familiar and useful AUT ideas, the average creativity score is 1.92 (min: 1.35, max: 2.59, SD=0.48), with an average novelty score of 1.74 (min:1.06, max: 2.76, SD=0.66), usefulness score of 4.36 (min: 3.47, max: 4.88, SD=0.55), and understandability score of 4.83 (min: 4.71, max: 5.00, SD=0.10); for the 10 novel but useless filled stimuli, the average creativity score is 2.45 (min: 2.00, max: 2.95, SD=0.28) and the average novelty score is 4.08 (min:3.65, max: 4.47, SD=0.27), the average usefulness score is 2.13 (min:1.65, max: 2.80, SD=0.36), and the average understandability score is 3.62 (min: 2.76, max: 4.35, SD=0.51). |

**Table S2 fMRI data acquisition and analyses in Detail**

| Section | Descriptions |
| --- | --- |
| MRI acquisition | The fMRI scanning was performed on a 3T Philips Achieva 3.0T TX MRI scanner with a 32-channel head coil at the Center for Biomedical Imaging Research, Tsinghua University. Functional images were acquired using a T2*-weighted echo-planar imaging sequence based on blood oxygenation level dependent (BOLD) contrast. The following acquisition parameters were used: TR = 2000 ms, TE = 30 ms, FOV = 200 mm × 200 mm, FA = 90°, 64 × 63 matrix, 30 slices, voxel size = 3.13 mm × 3.13 mm × 3.0 mm. High-resolution structural T1-weighted brain images were acquired for each participant (TR = 7.56 ms, TE = 3.70 ms, FOV = 256 mm × 256 mm, FA = 8°, 160 slices, voxel size = 1.0 mm × 1.0 mm × 2.0 mm). |
| Imaging data preprocessing | The imaging data were analyzed using SPM12 software. For preprocessing, the images for each subject were corrected for slice acquisition timing and realigned for head motion correction. And then the images were spatially normalized to a standard EPI template within SPM, and smoothed with a 6-mm full-width at half-maximum Gaussian kernel. |
| Univariate GLM analysis | The effects were estimated using the General Linear Model (GLM). At the first level analysis, two regressors of interest were defined as the trials in each condition: high conceptual expansion and low conceptual expansion, and the two regressors were modeled and convolved with the canonical hemodynamic response function (HRF). Then an additional regressor was modeled for filled trials that were out of interest, and six motion parameters were also included to control for head movement-related variability. Linear contrasts were used to obtain specific condition effects for each participant.  For the second-level analysis, the resulting contrast parameter estimate images were submitted to a random effects model, using paired T-Test to examine the effect of creative conceptual expansion. For the whole-brain analyses, a voxelwise threshold of *p* < .005 (uncorrected) was used and 30 or more contiguous voxels were reported. Furthermore, the middle temporal gyrus (MTG), which was predicted to be involved in creative conceptual expansion, would be chosen as region of interest (ROI). And the percentage signal changes within the defined ROIs were extracted for each condition separately for each participant using MarsBar. |

**Table S3** Brain regions associated with high conceptual expansion and low conceptual expansion.

| Brain regions | Hemisphere | Brodmann's area | MNI Coordinates | | | T | K |
| --- | --- | --- | --- | --- | --- | --- | --- |
|  |  |  | x | y | z |  |  |
| **High conceptual expansion > Low conceptual expansion** | | | | | | | |
| Hippocampus | right | - | 34 | -34 | -4 | 4.48 | 40 |
| Middle Temporal/Occipital Gyrus | right | 39 | 42 | -76 | 16 | 3.31 | 30 |
| Parahippocampal Gyrus | left | 37 | -32 | -40 | -4 | 4.85 | 103 |
| **Low conceptual expansion > High conceptual expansion** | | | | | | | |
| Medial Frontal Gyrus | left | 8 | -4 | 30 | 46 | 5.27 | 325 |
| Middle Frontal Gyrus | right | 9 | 54 | 18 | 36 | 3.72 | 161 |
| Middle Frontal Gyrus | left | 9 | -34 | 8 | 34 | 4.08 | 175 |
|  |  | 6 | -38 | 0 | 48 | 3.94 | 32 |

Threshold of voxel levels: T=2.76, *p*<0.005 (uncorrected), Cluster size is represented by k (k=30).
